# Menstrual Hygiene Management and School Absenteeism among Adolescents in Ghana: Results from a School-Based Cross-Sectional Study in a Rural Community

**DOI:** 10.1155/2020/6872491

**Published:** 2020-04-27

**Authors:** Shamsudeen Mohammed, Roderick Emil Larsen-Reindorf, Issahaku Awal

**Affiliations:** ^1^Department of Population, Family and Reproductive Health, School of Public Health, Kwame Nkrumah University of Science and Technology, Kumasi, Ghana; ^2^Department of Obstetrics & Gynaecology, School of Medical Science, Kwame Nkrumah University of Science and Technology, Kumasi, Ghana; ^3^Department of Medicine, Renal Dialysis Unit, Tamale Teaching Hospital, Tamale, Ghana

## Abstract

The study aimed to deepen our understanding of the menstrual hygiene management (MHM) of adolescents and the influence of menstruation on school absenteeism. We employed a school-based cross-sectional design in five Junior High Schools combining both quantitative and qualitative data collection methods. A questionnaire was used to collect quantitative data from 250 schoolgirls, and key informant interviews were conducted with a teacher in each of the five schools. We performed logistic regression analysis to provide crude and adjusted effect estimates and 95% confidence intervals. About fifty percent of the girls were engaged in good MHM, and approximately forty percent of them reported menstrual-related school absenteeism. We did not find evidence (*p* = 0.858) of association between MHM and menstrual-related school absenteeism. However, after controlling for the effect of other factors, we found evidence that the age of the schoolgirls, their father's occupation, and the receipt of allowance for menstrual care products were associated with MHM. When compared to those aged 17 to 19, those aged 10 to 13 years had 0.72 (95% CI 0.21, 2.44) decreased odds of poor MHM while those aged 14 to 16 had almost 3-fold increased odds (95% CI 1.49, 4.55) of poor MHM. The adolescents whose fathers were farmers had 0.42 (95% CI 0.21, 0.82) decreased odds of poor MHM while those whose fathers were unemployed had 0.24 (95% CI 0.10, 0.61) decreased odds of poor MHM. We found that girls who did not receive regular allowance for menstrual care products had nearly 2-fold increased odds (95% CI 1.06, 3.09) of poor MHM compared to those who received allowance for menstrual care products. Menstrual pain (82.2%), fear of staining clothing (70.3%), fear of being teased (70.3%), nonavailability of sanitary pad (63.4%), and lack of private place to manage period at school (60.4%) were the common reasons cited for menstrual-related school absenteeism.

## 1. Background

Menstruation is a natural part of the reproductive cycle during which blood is lost through the vagina [1]. Most adolescents experience their first menstruation (menarche) between the ages of 11 and 14 years. However, some girls start as early as 8 and some at 17 or older [2, 3]. Hygiene during the period of menstruation is fundamental to the dignity and well-being of women and girls and an issue that every girl has to deal with once she enters adolescence around the age of 12 and until she reaches menopause [4, 5]. Menstrual hygiene products such as tampons, sanitary pads, menstrual cups, cloths, paper material, or plant material are used by women and girls to absorb menstrual blood and to maintain personal hygiene during the period of menstruation [6]. To prevent physical discomfort and leakages, adolescent girls need to know the types of menstrual care products that exist and how to appropriately use and dispose of them hygienically [7]. Knowledge of what product to use, how often to change it, and access to adequate water, sanitation, and hygiene facilities help women and girls to maintain good hygiene during menstruation [1, 8]. In the absence of suitable and affordable menstrual care products, some women and girls may resort to using unhygienic and inappropriate products such as newspaper, old rags, dried leaves, or socks to collect menstrual blood and manage their periods [1, 8]. For instance, a study in Mali reported the use of old cotton fabric (*pagnes*) to absorb menstrual blood among adolescent girls [9]. Also, findings from a study in Ethiopia showed that 35.4% of students used sanitary napkins, 55.6% used homemade cloth, and 9% used underwear as absorbent materials for menstrual blood [10]. Although research on the health risks of poor menstrual hygiene practices is inconclusive [3], it is believed that hygiene practices during menstruation have a health impact as the risk of reproductive and urinary tract infections is higher than normal during menstruation [1]. Poor, unhygienic, and unsafe sanitary materials to absorb menstrual blood, therefore, predispose adolescent girls to reproductive tract infections with potential long-term effects on their reproductive health [11]. UNICEF suggests that, during menstruation, girls and women need to change their sanitary napkins regularly, especially in the first two to three days [5]. Sommer and Connolly proposed that menstrual absorbent products should change three or four times a day and woman and girls should wash the area around the vagina at least twice a day with soap and water [2].

Globally, menstruation and menstrual hygiene insecurity contribute to school absenteeism of millions of girls and women and increase the likelihood of school dropout [12]. According to UNESCO, many adolescent girls stay home from school due to menstrual cramping, deficient menstrual hygiene materials, lack of water and sanitation facilities in schools, unsupportive environments, and fear of a menstrual accident [7]. Some adolescents also shun standing up to answer questions in class because of fear of leakage or smell and discomfort; others hesitate to write on the board for fear of menstrual accidents and others seeing blood on their clothes and the subsequent shame and embarrassment this causes [13–16]. Tegegne and Sisay found that girls sometimes miss exams in school when the date of the exam coincides with their menstruation days [10]. This limits the right of women and girls to get an education and contribute to maintaining gender inequalities between schoolgirls and their male peers [12]. In some cases, girls completely drop out of school because of the stress and embarrassment associated with managing menstruation in a school environment [1]. With accessible water and adequate sanitation facilities that are safe, socially and culturally acceptable within the school premises, women and girls are able to manage menstruation in privacy and with dignity and do not have to miss out on their studies when they menstruate [12]. This study was undertaken to deepen understanding of the menstrual hygiene management of adolescents and the impact of menstruation and menstrual hygiene practices on school absenteeism among adolescent schoolgirls in a rural community in Ghana.

## 2. Materials and Methods

This was a school-based cross-sectional study conducted in Kumbungu in the Northern Region of Ghana. Kumbungu is the capital of the Kumbungu District in the Northern Region of Ghana with an estimated population of 39,341 [17]. All the people in this district live in rural areas with the population equally distributed between males and females [17]. There are five main Junior High Schools in the district capital. Adolescent schoolgirls in all the five Junior High Schools in the district constituted the population for the study, and data were collected from Junior High Schoolgirls between the ages of 10 and 19 years who had attained menarche. Participants were selected from Junior High Schools because girls experience menarche between 11 and 14 years and during this period they are in Junior High School.

### 2.1. Sample Size and Sampling Procedure

The sample size for the study was determined using Cochran's correction formula for a finite population [18] based on 40% prevalence of menstrual hygiene practices from a previous study [19], 95% confidence interval, and 5% margin of error. With the population of 387 adolescent schoolgirls in the five Junior High Schools and the assumption of 30% nonresponse rate, the calculated sample size for the study was estimated to be approximately 250.

The estimated sample size was apportioned to each school using probability proportional to size sampling approach centred on the number of adolescent girls in each of the five schools. A sampling frame of eligible participants for each school was designed with the help of schoolteachers using class registers. A list of random numbers was then generated and compared to the sampling frame to select participants through simple random sampling method. Using the classroom registers, selected students were identified and approached in their respective classrooms in each of the five schools. They were then put together in one classroom for completion of the study questionnaire. A teacher was purposively selected in each of the five schools for key informant interview (KII) on the impact of menstruation on school attendance. We selected classroom teachers who taught in their school for at least a year and were willing to consent to participate in the study, and female teachers who met the criteria were selected over male teachers because adolescent girls are more likely to disclose their menstrual challenges to them.

The research team then generated a table of random numbers with OpenEpi version 3, which was compared to a sampling frame to sample participants for the study in each of the five schools using simple random sampling method.

### 2.2. Data Collection Procedure

Quantitative data were collected with a pretested self-administered questionnaire consisting of both opened-ended and closed questions on sociodemographic characteristics of the participants, menstrual hygiene management, and the influence of menstruation on school attendance of adolescent girls. The questionnaire was field-tested among 30 adolescent girls with similar characteristics as the study population. The field test offered an opportunity to assess the reliability of the instrument and the ability of the participants to understand and respond to the questions. Necessary changes were made to improve the questionnaire after the review and the field test. Three female research assistants were recruited and trained to administer the questionnaire. With the assistance of teachers in each of the schools, participants were positioned apart from each other in classrooms to prevent discussions between them during completion of the questionnaire. Due to the culturally sensitive nature of menstruation and its management, adolescent boys and male teachers were not allowed in classrooms where quantitative data collection took place. The female research assistants explained to the participants how to complete the questionnaires and offered help to those who required it.

In addition, key informant interviews were conducted with a teacher in each of the five schools on the impact of menstruation on the school attendance of the adolescent schoolgirls. Trained research assistants used an interview guide to facilitate the key informant interviews. The questions were open-ended with probes, to help facilitators ask for more details where necessary. All the interviews were conducted in English language and recorded with an audio tape recorder with notes taken. The participants were informed of the purpose of the study and their right to decline to answer any question or withdraw from participation at any time during the study.

### 2.3. Definition of Terms

#### 2.3.1. Menstrual Hygiene Management

The study adopted the menstrual hygiene management definition proposed by the World Health Organization and UNICEF as the “use of clean menstrual management material to absorb or collect menstrual blood that can be changed in privacy as often as necessary for the duration of a menstrual period, using soap and water for washing the body as required, and having access to safe and convenient facilities to dispose of used menstrual management materials” [13].

#### 2.3.2. Menstrual-Related School Absenteeism

This was defined as missing one or more days of school because of menstruation or its management.

#### 2.3.3. Adolescent

Schoolgirls between the ages of 10 and 19 years were considered as adolescents in this study.

### 2.4. Data Management and Statistical Analysis

Data were checked for completeness, coded, and entered into Microsoft Excel Spreadsheet before imported into Stata version 16.0 for analysis. Background characteristics of the schoolgirls were summarised by whether they ever experienced menstrual-related school absenteeism. The adolescents' menstrual hygiene management was measured out of nine menstrual hygiene management questions and scored based on the scoring scheme of a previous study [19]. Each correct response scored one point while an incorrect response earned no score. The scores of participants were summed to determine their overall menstrual hygiene management score. Participants were classified as engaged in good menstrual hygiene management if they scored 6-9 points and poor menstrual hygiene management if they scored 0-5 points. We performed univariate and multivariable logistic regression analysis to provide crude and adjusted effect estimates and 95% confidence intervals for the association between sociodemographic factors and the menstrual hygiene management of the schoolgirls. Independent factors that showed strong or statistical significant (*p* < 0.05) effect in the initial univariate analysis were moved to the multivariable regression model where the effect of other factors was controlled to determine the factors that were independently associated with the menstrual hygiene management of the schoolgirls. Variables were only retained in the multivariable logistic regression model if they showed at least good evidence of association (*p* < 0.05). All *p* values presented are from the likelihood ratio test. Qualitative data from key informant interviews were transcribed verbatim. The transcripts were read several times to generate codes, which were then organised into one major theme, and narrated with quotations from the participants.

### 2.5. Ethical Consideration

The ethics committee of Kwame Nkrumah University of Science and Technology, School of Medical Sciences, and Komfo Anokye Teaching Hospital provided ethical clearance for the study, and permission was obtained from the Kumbungu District Director of Education. Participants indicated their consent by signing a consent form designed for the study after the purpose and significance of the study was explained to them. For participants under the age of consent, informed verbal consent was obtained from headteachers of their respective schools and signed informed assent from the participants. Parental consent was not obtained since headteachers act as guardians in school. The consent procedure was approved by the ethics committee and accepted by the headteachers. Confidentiality of the information collected was ensured.

## 3. Results

### 3.1. Sociodemographic Characteristics of Adolescent Schoolgirls

A little over half of the participants (*n* = 137, 54.8%) were 14 to 16 years old, and sixty-eight percent (*n* = 171) of them experienced menarche between 13 and 15 years (Table 1). Of the two hundred and fifty participants, 244 (97.6%) were Muslims, 132 (52.8%) lived in families that owned radio/television, and 66 (26.4%) of them were engaged in income-earning activities after school hours. More than half (*n* = 145, 58.2%) of the participants said they do not receive money for the purchase of menstrual care products.

### 3.2. Menstrual Hygiene Management of Adolescent Schoolgirls

Less than half (*n* = 113, 45.2%) of the schoolgirls changed their used absorbent materials three times or more in a day, and seventy-six percent (*n* = 190) of them reported that they wash their hands before changing their pads (Table 2). A little over half (*n* = 104, 54.74%) of the girls who washed their hands before changing their pads reported doing so always. The results showed that almost all (*n* = 237, 94.80%) the girls washed their hands after changing their used absorbent materials, and more than half (*n* = 168, 70.89%) of them do so always. Forty-five percent (*n* = 113) of the girls reported that they disposed of their used sanitary pads in latrine (pit latrine). One hundred and sixty-two (64.80%) of the girls cleaned their genitals with water and soap when menstruating, and almost all the girls (*n* = 235, 94.38%) bath during menstruation. Based on the overall menstrual hygiene management score, 127 (50.80%) of the participants were engaged in good menstrual hygiene management while 123 (49.20%) were engaged in poor menstrual hygiene management.

### 3.3. Factors Associated with Menstrual Hygiene Management of Adolescent Schoolgirls

In the bivariate analysis, independent factors that were associated with menstrual hygiene management (MHM) were the age of the schoolgirls, their father's occupation, and receipt of money for sanitary products (Table 3).

In the adjusted analysis, there was very strong evidence (*p* < 0.001) of association between age and poor menstrual hygiene management of the schoolgirls (Table 3). Compared to those aged 17 to 19, those aged 10 to 13 years had 0.72 (95% CI 0.21, 2.44) decreased odds of poor menstrual hygiene management while those aged 14 to 16 had almost 3-fold increased odds (95% CI 1.49, 4.55) of poor menstrual hygiene management. We found strong evidence (*p* = 0.005) that father's occupation was associated with the menstrual hygiene management of adolescents. Those whose fathers were farmers had 0.42 (95% CI 0.21, 0.82) decreased odds of poor menstrual hygiene management whereas those whose fathers were unemployed had 0.24 (95% CI 0.10, 0.61) decreased odds of poor menstrual hygiene management compared to those whose fathers had other forms of occupation. The results show good evidence (*p* = 0.028) of association between receipt of regular allowance for sanitary products and the menstrual hygiene management of the schoolgirls. Schoolgirls who did not receive regular allowance for sanitary products had nearly 2-fold increased odds (AOR 1.81, 95% CI 1.06, 3.09) of poor menstrual hygiene management compared to those who received an allowance for sanitary products.

### 3.4. Influence of Menstruation on the School Attendance of Adolescent Girls

Of the two hundred and fifty schoolgirls, 101 (40.4%) ever absented from school because of menstruation (self-reported); the rest (*n* = 149, 59.6%) of the girls said they have never missed school because of menstruation. Less than half of the girls who were engaged in good menstrual hygiene management (*n* = 52, 40.9%) and 49 (39.8%) of those engaged in poor menstrual hygiene management reported menstrual-related school absenteeism (Table 3). However, we did not find any evidence (*p* = 0.858) of association between menstrual hygiene management and menstrual-related school absenteeism. The most frequently reported reason for missing school among the one hundred and one respondents who experienced menstrual-related absenteeism was pain (*n* = 83, 82.2%) followed by fear of staining clothing (*n* = 71, 70.3%), fear of being teased (*n* = 71, 70.3%), nonavailability of sanitary pad (*n* = 64, 63.4%), and lack of private place to manage period at school (*n* = 61, 60.4%) (Table 4).

### 3.5. Schoolteachers' Perspective on Menstrual-Related Absenteeism

Almost all the teachers we interviewed said menstruation does not affect the school attendance of most of the girls in the schools. However, they disclosed that some girls occasionally ask for permission from teachers to go home and manage severe menstrual symptoms (e.g., menstrual pains). One teacher said some girls come to school late when they are menstruating. A teacher mentioned that they do not advise girls to absent from school when they are menstruating “because menstruation is not a disease and lessons will not be revisited when they resume school after their periods.” The teachers, however, stated that menstruation negatively influences the participation of girls during lessons. Some of the teachers cited absenteeism, poor concentration in class, and loss of self-confidence during the period of menstruation as some of the factors that may impact negatively on the academic performance of girls.

*It doesn't affect their attendance that much because most of them come to school when they are menstruating and at times they do ask for permission to either absent or leave early under the excuse of headache and or abdominal pains (KII, male teacher, Kumbungu)*.

*Yes, menstruation affect their class participation in the sense that, they always become timid and at times their mood in the class changes; they tend not to be active and appear moody and do not participate or get involved in class activities which directly affect their end of term results (KII, female teacher, Kumbungu)*.

*Some of them tell you they are sick because they experience headache, abdominal pains, dizziness, nausea which affect their class participation, their confidence in class and even their movement in the class since they don't want to get soiled by the blood (KII, female teacher, Kumbungu)*.

## 4. Discussion

We found that approximately half of the girls were engaged in good menstrual hygiene management and about forty percent of them reported menstrual-related school absenteeism. After controlling for the effect of other factors, there remained evidence that the age of the schoolgirls, their father's occupation, and receipt of allowance for menstrual care products were associated with their menstrual hygiene management. Evidence shows that menstrual hygiene practices vary between girls of different ages [20] and this may explain why girls between the ages of 14 and 16 had almost 3-fold increased odds of poor menstrual hygiene management compared to those in the older age group. This is perhaps due to older girls' better knowledge about menstrual products, its use, and storage as they would have had more opportunity to learn from their peers and family compared to younger girls. A study in India found that older girls use more appropriate menstrual care products, change absorbent materials more frequently, and bath frequently during menstruation [20].

Our findings showed that most of the girls who were engaged in good hygiene practices received allowance from parents for menstrual care products, and girls who did not receive regular allowance for sanitary products were nearly 2 times more likely to engage in poor menstrual hygiene management compared to those who received allowance for sanitary products, which is consistent with similar studies in Ethiopia [19] and India [20]. In low- and middle-income settings, the cost of sanitary products is one of the major barriers to good hygiene practices during menstruation. It has been observed that family socioeconomic status influence girls' choice of menstrual care products, and this may further explain the association between fathers' occupation and the menstrual hygiene management of the girls. A study in India reported that girls from low socioeconomic families use unsanitary or substandard menstrual absorbents and lack access to hygiene facilities to manage menstruation [20].

A greater number of the girls in this study used commercial sanitary pads as compared to other menstrual absorbent materials during their last period before this study, which is contrary to the findings of studies in Ethiopia [10] and Mali [9] where adolescent girls used clothes, old cotton fabric, and rags as absorbent materials. The use of proper menstrual absorbent material will probably reduce the feeling of discomfort, prevent leakages and stains, and reduce their risk of infection during menstruation. However, only 45.2% of the girls changed their menstrual pads three or more times in a day, which is inconsistent with a study in Senegal where more than half (54.7%) of the study participants changed their menstrual absorbent materials at least three times in a day [21]. Not changing pads frequently enough, especially if they are inserted into the vagina, can introduce or support the growth of unwanted bacteria [3]. In India, Torondel et al., found that frequent changing of absorbent material was protective against bacterial vaginosis [22].

Sommer and Connolly encourage girls and women to wash the area around the vagina at least twice a day with mild soap and plain water when menstruating [2]. This practice was reported in nearly two-thirds of the schoolgirls in this study. Frequent washing of the genitals during menstruation reduces the incidence of reproductive tract infections. A study in India reported bacterial vaginosis and candida infection in women who practiced lower frequency of personal washing during their periods [22].

Consistent with the findings of a study in Zambia [23], many of the girls in this study were not engaged in good disposal practices as the majority of them disposed of their used absorbent materials in toilets (pit toilets). This method of disposal may be preferred because girls do not want others to see their used pads and probably because of the lack of dustbins in school toilets. In Zambia, Chinyama et al. found that girls disposed of their used pads in latrines because they were afraid their used pads could be used for witchcraft against them [23]. Nonetheless, improper disposal of blood-stained menstrual absorbent materials can contaminate the environment and spread diseases [4].

Menstrual-related school absenteeism was reported in less than half of the girls in this study, which is in contrast with similar studies in Ethiopia [10] and Uganda [24] but consistent with studies in Indonesia [15], South Africa [25], and Bangladesh [26]. Schoolteachers revealed that girls who attend school when menstruating feel shy to participate in school activities and exhibit poor concentration in class and loss of self-confidence. Several studies in low- and middle-income countries reported similar findings on the participation of girls in school activities during their periods [14–16]. In Nepal, Morrison et al. found that during their period of menstruation most girls are uncomfortable sitting near the front of the classroom and would never raise their hand to answer questions or write on the board [16]. The most frequently reported reasons for menstrual-related absenteeism in this study were menstrual pain, fear of staining clothing, and fear of being teased by colleagues, which are similar to findings reported in earlier studies [14–16]. These concerns together with the occasional school absenteeism may cause some girls to lag in their curricular activities particularly in complex and abstract subjects and may eventually lead to dropout [27]. The decreased attention span and poor concentration among adolescent schoolgirls during menstruation may be linked to the discomfort associated with menstruation, particularly dysmenorrhoea and excessive bleeding, as well as the mental stress during menstruation, mainly due to constant worry that others may know about their menstruation [28]. Education, undoubtedly, is a means of empowering women and girls, and for this reason, the barriers preventing women and girls from realising their right to education must be acknowledged and fully eliminated [12].

## 5. Limitations

The study has several limitations that should be considered when interpreting the findings. Firstly, data on school facilities, household income, parental education, and other potential risk factors for poor menstrual hygiene management were not collected. Data on these factors could have provided a deeper understanding of how girls manage menstruation in school, whether there was a supportive school environment and how household income influences the hygiene practices of girls. Secondly, this was a cross-sectional study and recall bias might have been introduced by the nature of the design. Thirdly, information about the class activities and school performance of the girls during menstruation were collected from classroom teachers and this could have been overstated. Finally, menstrual hygiene practices were self-reported by the adolescents, and reporting bias cannot be ruled out even though efforts were made to minimise it.

## 6. Conclusion

Approximately half of the girls were engaged in good menstrual hygiene practices, and about forty percent of them reported menstrual-related school absenteeism. We found evidence that the age of the schoolgirls, their father's occupation, and receipt of allowance for menstrual care products were associated with their menstrual hygiene management. Further research on the impact of menstruation and its management on the academic performance of adolescent schoolgirls is recommended. The study further recommends large-scale studies on the reproductive health effects of poor menstrual hygiene practices to fully understand the effects of menstrual hygiene management beyond school attendance and academic performance.

## Figures and Tables

**Table 1:** Sociodemographic characteristics of adolescent schoolgirls.

Characteristics	Menstrual-related school absenteeism		Total (%)
	Yes (%)	No (%)	
Age group (years)			
10-13	7 (6.93)	9 (6.04)	16 (6.40)
14-16	50 (49.50)	87 (58.39)	137 (54.80)
17-19	44 (43.56)	53 (35.57)	97 (38.80)
Age at menarche			
10-12	27 (26.73)	46 (30.87)	73 (29.20)
13-15	70 (69.31)	101 (67.79)	171 (68.40)
16-17	4 (3.96)	2 (1.34)	6 (2.40)
Have television/radio			
Yes	57 (56.44)	75 (50.34)	132 (52.80)
No	44 (43.56)	74 (49.66)	118 (47.20)
Earn money			
Yes	29 (28.71)	37 (24.83)	66 (26.40)
No	72 (71.29)	112 (75.17)	184 (73.60)
Father's occupation			
Trader	5 (4.95)	11 (7.38)	16 (6.40)
Farmer	71 (70.30)	87 (58.39)	158 (63.20)
Formal sector employee	1 (0.99)	14 (9.40)	15 (6.00)
Unemployed	14 (13.86)	24 (16.11)	38 (15.20)
Others^a^	10 (9.90)	13 (8.72)	23 (9.20)
Mother's occupation			
Trader	23 (22.77)	35 (23.49)	58 (23.20)
Farmer	33 (32.67)	37 (24.83)	70 (28.00)
Formal sector employee	6 (5.94)	15 (10.07)	21 (8.40)
Unemployed	28 (27.72)	51 (34.23)	79 (31.60)
Others^a^	11 (10.89)	11 (7.38)	22 (8.80)
Receive money for sanitary pad			
Yes	38 (38.00)	66 (44.30)	104 (41.77)
No	62 (62.00)	83 (55.70)	145 (58.23)
Summary of menstrual hygiene management^b^			
Good MHM	52 (40.9)	75 (59.06)	127 (50.8)
Poor MHM	49 (39.8)	74 (60.2)	123 (49.2)

^a^Others include tailor, daily labourer, butcher, imam, and mason. ^b^Chi-squared test: *x*^2^ = 0.0318, *p* value = 0.858. MHM: menstrual hygiene management.

**Table 2:** Menstrual hygiene management of adolescent schoolgirls.

Parameters of practice	Number	Percent
Absorbent material used during the last menstrual period		
Commercial sanitary pad	118	47.20
Reusable cloth pad	85	34.00
Disposable cloth	25	10.00
Cotton	7	2.80
Underwear/underpants	15	6.00
Change of absorbent material		
Once daily	28	11.20
Twice daily	101	40.40
Thrice or more daily	113	45.20
Do not change until the next day	8	3.20
Wash hands before changing pad		
Yes	190	76.00
No	60	24.00
How often do you wash your hands before changing your pad (*n* = 190)		
Always	104	54.74
Most of the time	54	28.42
Sometimes	32	16.84
Wash hands after changing pad		
Yes	237	94.80
No	13	5.20
How often do you wash your hands after changing your pad (*n* = 237)		
Always	168	70.89
Most of the time	38	16.03
Sometimes	31	13.08
Disposal of used absorbent materials		
Dustbin	38	15.20
Toilet	113	45.20
Open environment	2	0.80
Burnt	25	10.00
Buried	72	28.80
Materials for cleaning genitals when menstruating		
Water only	71	28.40
Water and soap	162	64.80
Tissue paper	8	3.20
Towel	3	1.20
I do not clean/wash	6	2.40
Bath during menstruation		
Yes	235	94.38
No	14	5.62
Overall menstrual hygiene practice		
Good practice	127	50.80
Poor practice	123	49.20

Note: all the menstrual hygiene practice questions were self-reported by the adolescents.

**Table 3:** Association of sociodemographic factors and menstrual hygiene management of adolescent schoolgirls.

Characteristics	Good (%) MHM	Poor (%) MHM	Crude odds ratio (95% CI)	*p* value^1^	Adjusted odds ratio (95% CI)	*p* value^1^
Age group (years)						
10-13	10 (62.50)	6 (37.50)	0.97 (0.33, 2.90)	0.006	0.72 (0.21, 2.44)	*p* < 0.001
14-16	57 (41.61)	80 (58.39)	2.28 (1.34, 3.88)		2.61 (1.49, 4.55)	
17-19	60 (61.86)	37 (38.14)	1		1	
Age at menarche						
10-13	63 (48.09)	68 (51.91)	1.26 (0.76, 2.07)	0.369		
14-17	64 (53.78)	55 (46.22)	1			
Father's occupation						
Other^2^	19 (35.19)	35 (64.81)	1	0.018	1	0.005
Farmer	84 (53.16)	74 (46.84)	0.48 (0.25, 0.91)		0.42 (0.21, 0.82)	
Unemployed	24 (63.16)	14 (36.84)	0.32 (0.13, 0.75)		0.24 (0.10, 0.61)	
Mother's occupation						
Other^2^	20 (46.51)	23 (53.49)	1	0.151		
Farmer	34 (48.57)	36 (51.43)	0.92 (0.43, 1.97)			
Trader	37 (63.79)	21 (36.21)	0.49 (0.22, 1.10)			
Unemployed	36 (45.57)	43 (54.43)	1.04 (0.49, 2.19)			
Have television/radio						
Yes	70 (53.03)	62 (46.97)	1			
No	57 (48.31)	61 (51.69)	1.21 (0.73, 1.99)	0.456		
Earn money for yourself						
Yes	37 (56.06)	29 (43.94)	1			
No	90 (48.91)	94 (51.09)	1.33 (0.76, 2.35)	0.319		
Receive regular money for menstrual care products						
Yes	61 (58.65)	43 (41.35)	1		1	
No	66 (45.52)	79 (54.48)	1.70 (1.02, 2.83)	0.041	1.81 (1.06, 3.09)	0.028

MHM = menstrual hygiene management; CI = confidence interval. ^2^Others include tailor, formal sector employee, daily labourer, butcher, imam, and mason. ^1^*p* values are from the likelihood ratio test.

**Table 4:** Adolescent schoolgirls' reasons for missing school during menstruation (*n* = 101).

Reasons^a^	Number	Percent (%)
Afraid of staining clothing	71	70.30
Afraid of being teased	71	70.30
Pain	83	82.18
Lack of private space to manage period at school	61	60.40
Lack of disposal facilities for used pad/cloth	50	49.50
I do not have a sanitary pad	64	63.37
Cultural restrictions	30	29.70
Religious restrictions	17	16.83
My parents asked me not to go to school	26	25.74
My teacher told me not to come to school	19	18.81

^a^ Multiple responses were allowed and the percentage is greater than 100.

## Data Availability

The data used to support the findings of this study are available from the corresponding author upon request.

## Abbreviations

KII: Key Informant Interviews
MHM: Menstrual Hygiene Management.
